# Significant Reduction in Vertebral Artery Dose by Intensity Modulated Proton Therapy: A Pilot Study for Nasopharyngeal Carcinoma

**DOI:** 10.3390/jpm11080822

**Published:** 2021-08-22

**Authors:** Yun-Hsuan Lin, Jen-Yu Cheng, Bing-Shen Huang, Sheng-Dean Luo, Wei-Che Lin, Shang-Yu Chou, Pei-Jiuan Juang, Shen-Hao Li, Eng-Yen Huang, Yu-Ming Wang

**Affiliations:** 1 Department of Radiation Oncology and Proton & Radiation Therapy Center, Kaohsiung Chang Gung Memorial Hospital, Chang Gung University College of Medicine, Kaohsiung 83301, Taiwan; er1122@cgmh.org.tw (Y.-H.L.); york480@cgmh.org.tw (J.-Y.C.); beanson@cgmh.org.tw (B.-S.H.); a9682@cgmh.org.tw (S.-Y.C.); eggq@cgmh.org.tw (P.-J.J.); leeshenhao@gmail.com (S.-H.L.); hey1200@cgmh.org.tw (E.-Y.H.); 2 Department of Radiation Oncology, Linkou Chang Gung Memorial Hospital, Chang Gung University College of Medicine, Taoyuan 33305, Taiwan; 3 Department of Radiation Oncology, Xiamen Chang Gung Hospital, Xiamen 361126, China; 4 Department of Otolaryngology, Kaohsiung Chang Gung Memorial Hospital, Chang Gung University College of Medicine, Kaohsiung 83301, Taiwan; rsd0323@cgmh.org.tw; 5 Department of Diagnostic Radiology, Kaohsiung Chang Gung Memorial Hospital, Chang Gung University College of Medicine, Kaohsiung 83301, Taiwan; alex@cgmh.org.tw; 6 School of Traditional Chinese Medicine, Chang Gung University College of Medicine, Taoyuan 33302, Taiwan

**Keywords:** IMPT, proton therapy, vertebral artery, stroke, carotid artery stenosis, nasopharyngeal carcinoma

## Abstract

Intensity modulated proton therapy (IMPT) with the vertebral artery (VA)-sparing technique has been initially proposed in our institution. This pilot study was conducted to compare the dose to VAs between IMPT and volumetric-modulated arc therapy (VMAT) for patients with nasopharyngeal carcinoma (NPC). A total of six patients with NPC treated by IMPT were enrolled in the study. Target volumes and organs at risk (OARs) were delineated, including 12 samples of right and left VAs, respectively, for each patient. Treatment planning by IMPT and dual-arc VMAT was carried out for comparison. The IMPT plan significantly reduced VA mean dose, V10, V20, V30, V40, and V50, compared to the VMAT plan in all 12 samples (*p* < 0.001). The average mean dose to VAs for IMPT was 35.2% (23.4–46.9%), which was less compared to VMAT (*p* < 0.001). Adequate dose coverage was achieved with both IMPT and VMAT plans for three different dose levels of target volumes for all patients. IMPT significantly reduces VA dose while maintaining adequate dose coverage of all target volumes. For patients with head and neck cancer who seek to preserve their blood flow to the brain in order to decrease late vascular and neurologic sequelae, IMPT should be considered. A prospective study with longer follow-up is ongoing to confirm our preliminary results.

## 1. Introduction

Nasopharyngeal carcinoma (NPC) is highly prevalent in East Asia [1]. Radiotherapy (RT) is the mainstay treatment for NPC and has resulted in excellent disease control [2,3]. However, late vascular injuries caused by RT, such as carotid artery (CA) stenosis, vertebral artery (VA) stenosis, and ischemic stroke, have raised significant concerns about cancer survivorship [4,5,6,7,8,9,10]. CA and VA stenosis, for example, has an incidence rate reaching 30–40%, and the incidence increases continually with longer intervals after RT [9,10]. These late vascular and neurologic sequelae not only negatively affect the quality of life after treatment but also increase the healthcare expenditure for these long-term survivors. Refinement of the RT treatment to reduce these late sequelae and, meanwhile, maintain excellent disease control is the goal of the radiation oncologists.

Proton beam therapy (PBT), with its physics advantage of Bragg peaks, has the benefit of dose distribution for cancer treatment but the reimbursement policy regarding PBT varies among different areas of the world. Recent advances in the delivery of PBT by intensity modulated proton therapy (IMPT) have been reported, with significant reductions in the dose to oral cavity, brainstem, spinal cord, whole brain, mandible, larynx, and esophagus, and acute toxicities, such as mucositis requiring gastrostomy tube insertion, over photon therapy (XRT) [11,12,13]. However, none of the previous studies investigated whether IMPT could reduce the dose to blood vessels with the aim of decreasing late vascular injuries.

The circulation of the human brain is supplied by the CAs and VAs. Since CAs lie inside the target volumes of patients with NPC, which could not be spared during RT treatment, IMPT with a VA-sparing technique has been proposed in our institution in order to achieve the goal of preserving cerebral blood flow and avoiding late vascular sequelae. This study was conducted as the first report of our VA-sparing IMPT and here we present our results comparing the dose to VAs between IMPT and volumetric-modulated arc therapy (VMAT).

## 2. Materials and Methods

### 2.1. Study Cohort

Under institutional review board approval, a total of 6 patients with non-distant metastatic NPC receiving IMPT at Kaohsiung Chang Gung Memorial Hospital were enrolled in this study. Patient charts were reviewed to determine patient characteristics and tumor characteristics. Their corresponding XRT plans were retrospectively generated using VMAT, the most advanced technique for XRT delivery, for comparison.

### 2.2. Simulation

All patients underwent computed tomography (CT) simulation in the supine position with a 1.25 mm slice thickness and were immobilized with custom thermoplastic masks. Contrast-enhanced CT and magnetic resonance imaging with and without gadolinium enhancement performed in the treatment position were used for image registration with the CT.

### 2.3. Target Delineation

The clinical target volumes were delineated based on published consensus [14]. Gross tumor volume (GTV) and three different dose levels of clinical target volumes (CTVs) were delineated. CTV_6996_ was defined as the GTV with an isotropic extension plus the entire nasopharynx. CTV_5940_ covered the areas at high risk for microscopic involvement, including the entire nasopharynx, posterior third of the nasal cavity and maxillary sinus, pterygoid plate, parapharyngeal space, retropharyngeal lymph nodes, clivus, skull base, inferior sphenoid sinus, and bilateral upper neck lymph nodes. CTV_5280_ encompassed bilateral lower neck nodes. Critical organs at risk (OARs) contoured on the CT images included brainstem, spinal cord, eye, lens, optic nerve, cochlea, parotid gland, submandibular gland, oral cavity, mandible, larynx, pharynx, cervical esophagus, and thyroid gland. Twelve samples of VAs, including right and left VAs, respectively, for each patient, were also delineated. Right and left VAs originated from right and left subclavian arteries separately, and terminated at the site they merged to form the basilar artery. To precisely delineate the VAs, rigid registration of the contrast-enhanced CT was performed for better visualization.

All contours in all cases were reviewed for quality assurance by experienced head and neck radiation oncologists before treatment planning. The same contours were used for the IMPT and VMAT.

### 2.4. Dose Prescription

The prescription doses were 69.96 Gy (RBE), 59.4 Gy (RBE), 52.8 Gy (RBE) at 2.12 Gy (RBE), 1.8 Gy (RBE), 1.6 Gy (RBE) per fraction to the high-risk, intermediate-risk, and low-risk target volumes, respectively, using the simultaneous integrated boost technique. The goals for target coverage and dose constraints to OARs were the same for IMPT and VMAT planning.

### 2.5. Treatment Planning

Treatment planning for IMPT and VMAT plans was carried out by RayStation (version 8.1, RaySearch Laboratories, Stockholm, Sweden). IMPT plans were created for full-field IMPT by using 3 fields from 3 different directions of scanning beams. Robust optimization was used to account for beam range uncertainties (±3.5%) and setup uncertainties (±3 mm). The treatment planning system simultaneously optimized the spot intensities from all fields, using an optimization algorithm with the objective of covering 99.5% of the corresponding CTVs with a 100% prescribed dose while minimizing dose to the adjacent OARs.

VMAT plans were designed using two full arcs. A 3 mm planning target volume (PTV) expansion was added to the three CTVs, respectively, for set-up uncertainties. The optimization objective covered 95% of the PTVs and 99.5% of the CTVs with a 100% prescribed dose. Both IMPT and VMAT plans were created by using identical dose–volume histogram objectives.

For VAs, there is currently no dose–volume consensus. The optimization goal that we proposed for our patients was a mean dose of 20 Gy (RBE) and V30 < 30% for both IMPT and VMAT. For comparisons of IMPT and VMAT, dose–volume parameters such as the mean dose, V10, V20, V30, V40, V50, V60, and V70 of VAs were generated by the treatment planning system. V10, for instance, represents the percentage of volume receiving 10 Gy (RBE) or more of doses.

### 2.6. Statistical Analysis

Descriptive statistics were used to summarize patient data and tumor characteristics. The respective dose–volume histograms of the VAs of the 12 samples and three different levels of CTVs were exported from the treatment planning system for dose and volume analysis. The paired *t*-test was used to estimate the statistical significance of dose differences to VAs and target volumes between IMPT and VMAT. The Statistical Package for Social Sciences version 22.0 software (SPSS, Chicago, IL, USA) was used for statistical processing.

## 3. Results

### 3.1. Patient and Tumor Characteristics

The age, T stage, N stage, AJCC stage group, primary tumor laterality, and involved regional lymph node distribution of the six patients are summarized in Table 1. Five patients were male and one was female. According to the 8th edition of the American Joint Committee on Cancer (AJCC) staging system, one patient had stage I disease, two patients had stage II disease, and three patients had stage III disease. The primary nasopharyngeal tumors were right-sided in three patients, left-sided in two patients, and central in one patient. The distribution of involved regional lymph nodes was right-sided in two patients, left-sided in two patients, and bilateral in one patient. Five patients received induction and concurrent chemotherapy.

### 3.2. Treatment Planning Comparison

#### 3.2.1. Vertebral Arteries

**Figure 1:**
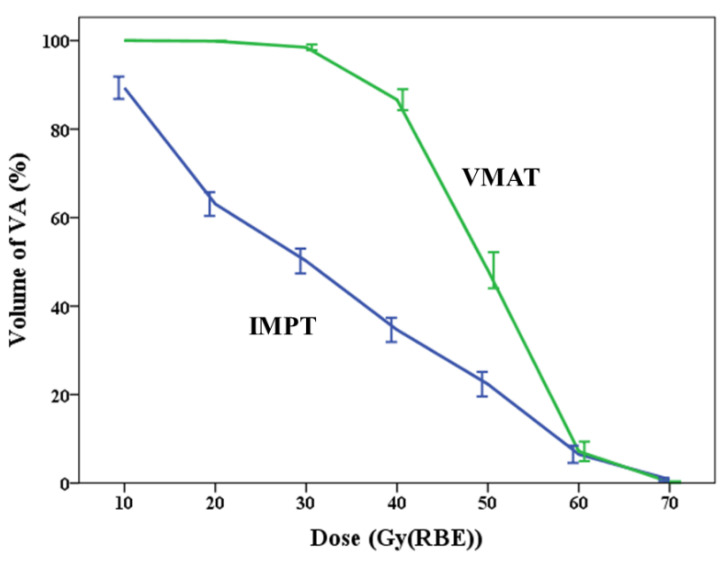
Differences in dose–volume indices of V10 to V70 to vertebral arteries between intensity modulated proton therapy (IMPT, blue line) and volumetric modulated arc therapy (VMAT, green line). (Error bar = ± 1 S.E).

The IMPT plan significantly reduced VA mean dose, V10, V20, V30, V40, and V50, compared to the VMAT plan in all 12 samples. The mean dose to VAs was 31.6 Gy (RBE) for IMPT and 48.79 Gy (RBE) for VMAT (*p* < 0.001). Though most of the VAs could not achieve the optimization goal that we proposed, the mean dose to VAs by IMPT was 35.2% less compared to VMAT. The comparisons of V10, V20, V30, V40, V50, V60, and V70, between IMPT and VMAT, are demonstrated in Figure 1. Table 2 depicts the detailed differences in doses to VAs between IMPT and VMAT.

**Figure 2:**
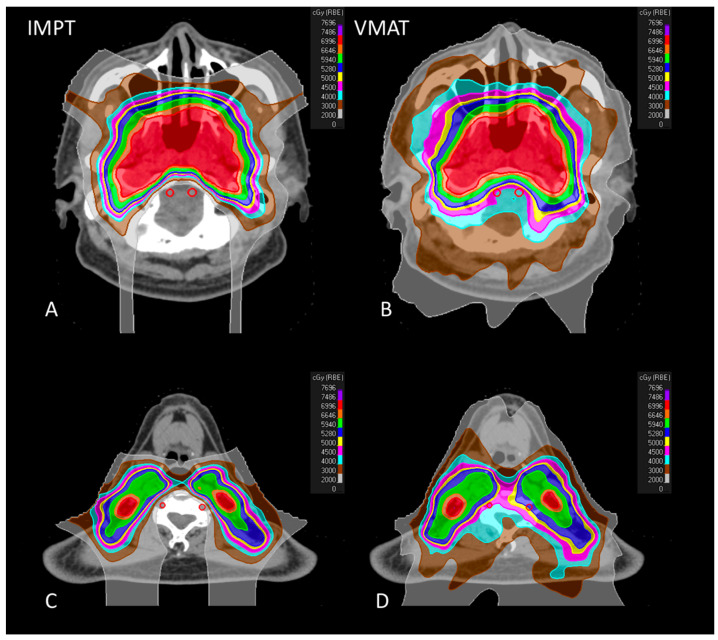
Representative axial CT images of colorwash dose distribution comparison for IMPT (left side) and VMAT (right side) plans of the same patient. (**A**): the dose distribution located at skull base level IMPT; (**B**): the dose distribution located at skull base level VMAT; (**C**): the dose distribution at the level of the fourth cervical vertebra of IMPT; (**D**): the dose distribution at the level of the fourth cervical vertebra of VMAT.

Figure 2 shows the representative axial CT images of colorwash dose distributions for IMPT and VMAT plans of the same patient.

#### 3.2.2. Target Coverage

Adequate dose coverage was achieved with both IMPT and VMAT plans for CTV_6996_ (IMPT, 99.5 versus VMAT, 99.8; *p* = 0.776), CTV_5940_ (IMPT, 99.8 versus VMAT, 99.9; *p* = 0.213), and CTV_5280_ (IMPT, 99.9 versus VMAT, 99.9; *p* = 0.091), for all patients. There was no significant difference between mean dose to CTV_6996_, although the mean doses to CTV_5940_ and CTV_5280_ were significantly higher with IMPT. The D95% and D5% were similar between IMPT and VMAT plans. The dose statistics of the coverage indices for CTV_6996_, CTV_5940_, and CTV_5280_ are demonstrated in Table 3.

## 4. Discussion

To the best of our knowledge, this is the first study in the world to raise the concept of VA sparing by IMPT and focusing on the benefit of IMPT to VAs in NPC patients. Our results demonstrated that the dose to VAs was significantly reduced in IMPT, even in comparison to the modern XRT technique of VMAT, while the coverage to the target volumes was equivalent and fulfilled the treatment requirement.

The long-term effect of ionizing radiation to cause vascular injury has been well studied [4,15,16]. This long-term change leads to sequelae such as CA stenosis, VA stenosis, ischemic stroke, transient ischemic attack, and neurocognitive functional loss [4,5,6,7,8,9,10]. CA and VA stenosis is a well-documented late complication for NPC patients treated with RT [4,6,7,8,9,10]. The incidence of CA and VA stenosis was 93.1% and 81.9%, respectively, observed by Zhou et al. after a median follow-up of 68 months after RT through using contrast-enhanced MR angiography; they also noted that the incidence of significant (>50%) CA and VA stenosis was 37.5% and 34.7%, respectively [10]. This prevalence is even more common with older age, higher RT dose, and longer duration after RT [9,10]. As for stroke, the 15-year cumulative risk of stroke after neck RT was 12.0% [17]. Stroke is a leading cause of mortality and serious long-term morbidity worldwide and stroke-related costs have kept rising; in the United States, for instance, the costs are over USD 46 billion per year [18]. Presently, with the improvement of treatment, more than 80% of NPC patients can achieve long-term survival [2,3]. These vascular and neurologic sequelae not only negatively affect the quality of life after treatment but also increase the healthcare expenditure for these long-term survivors. If late vascular sequelae could be decreased by sparing the related major blood vessels, quality of life of the patients would improve, and the healthcare expenditure would be reduced.

The benefit on dose distribution of PBT over XRT has been reported in previous studies, and several previous studies also reported early outcomes of IMPT for NPC [11,12,13]. Holliday et al. reported that patients treated with IMPT had significantly lower mean doses to the oral cavity, brainstem, whole brain, and mandible, and decreased rates of gastrostomy tube placement [11]. Lewis et al. showed that the mean dose received for structures including the cochlea, the vestibules of the ear, esophagus, larynx, mandible, oral cavity, tongue, area postrema, subthalamic nucleus, spinal cord, and whole brain was lower with IMPT compared to intensity modulated RT (IMRT) and no patients developed grade 3 body weight loss after IMPT [12]. Nevertheless, none of the past studies have investigated the feasibility of IMPT to spare the blood vessels with the aim of decreasing late vascular injuries.

For the CAs, in early glottic cancer, IMRT or VMAT makes it possible to create plans with steep dose gradients between the larynx CTV and the CAs, thus reducing the dose to the CAs [19,20]. Yet, this technique is not feasible for treatment of NPC. The consensus on target volumes of RT among patients with NPC has been well established [14,21]. Based on the consensus, it is necessary to irradiate the CAs as they are just inside the nodal CTVs, thus limiting the possibility to reduce the dose to CAs in our study [6]. In our study, the mean dose to CAs was around 100% of the prescribed dose to the corresponding CTVs. In fact, techniques trying to spare CAs or to reduce the dose to CAs should be weighted with the risk of locoregional recurrence for NPC patients.

However, techniques trying to spare VAs are another story. Since VAs are not directly inside the CTVs for NPC, it is not necessary to irradiate the VAs to the corresponding target dose so efforts to reduce doses to VAs should be encouraged. With the distance between the VAs and the CTVs, any techniques that could deliver steep dose distribution between these regions could theoretically reduce the risk of late injury to the VAs while maintaining the same locoregional control rate equivalent to XRT. With the physics advantage of PBT and the use of IMPT, sparing the VAs could be achievable. In our study, the dose to VAs by IMPT was 35.2% less compared to VMAT which both included strict dose constraints. This means the risk of vascular injury could be largely reduced with IMPT.

The blood supply of the brain comes mainly from the CAs, and the VAs serve as collateral supply from the circle of Willis. Although previous researchers investigating vascular injury mainly focused on CAs, VAs contribute 25–30% of blood to the brain [22], and approximately 20–25% of all acute strokes occur in the posterior circulation [23]. At the present time, under routine RT treatment by XRT using IMRT or VMAT techniques, VAs are usually exposed to a moderately high dose. The data of the dose–response threshold for radiation effects for VAs are lacking in the literature. For the CAs, Martin et al. have reported that there is a significant dose–response threshold of 35–50 Gy for radiation effects on the intimal–medial thickness of CAs [24]. For heart and coronary arteries, rates of major coronary events after RT for breast cancer increased linearly with the mean dose to the heart by 7.4% per Gray, and there was no apparent threshold noted for breast cancer; the increase started within the first 5 years after RT, and continued for at least 20 years [25,26]. Using IMPT, the mean dose to VAs was largely reduced from 48.79 Gy (RBE) of the VMAT plans to 31.6 Gy (RBE), which has the potential to decrease the risk of vascular sequelae.

More importantly, several investigators have observed that blood flow in VAs increases to compensate for the decreased brain perfusion in those with unilateral/bilateral CCA/ICA stenosis [27,28,29,30]. Thus, in theory, reducing the VA dose through IMPT to prevent related vascular injury may decrease the risk of developing sequelae related to brain circulation, since the ability of VAs to generate collateral circulation pathways is maintained as much as possible. With the potential of long-term survival, the high incidence of late CA stenosis, and the high cost of healthcare expenditure for patients with cerebrovascular accident, the use of VA-sparing IMPT should be advocated for patients with NPC.

There are several limitations in our study. First, the study patient numbers are limited. Since the cost of PBT is not reimbursed by our national health insurance, it can only be applied to a limited number of patients who are able to afford it. As this study was set up as a pilot study, we retrospectively generated only six patients who were treated by IMPT with their corresponding VMAT plan for analysis. Second, the constraint we initially set for VAs could not be achieved in most patients we treated. With additional patients and with longer follow-up, we will examine the relationship between the dose, irradiated volumes, and the residual flow of VAs as well as the related vascular and neurologic sequelae. In addition, longer follow-up will enable us to evaluate the vital issue of whether the outcome regarding tumor control in the patients treated with VA-sparing IMPT we have described is comparable to the outcome of patients treated with the current standard technique.

## 5. Conclusions

VA-sparing IMPT significantly reduces VA dose compared to VMAT while maintaining adequate dose coverage of all target volumes. For patients with head and neck cancer who seek to preserve the blood flow of the brain in order to decrease late vascular and neurologic sequelae, IMPT should be considered. In our institution, a prospective study with long-term follow-up is ongoing to confirm the benefit.

## Figures and Tables

**Table 1:** Patient characteristics and tumor characteristics.

Patient	Age	Sex	WHO Type	T Stage	N Stage	Clinical Stage	Tumor Laterality	Lymph Node Distribution
1	26	Male	3	3	1	III	Right	Right RP, level Va
2	56	Male	3	1	1	II	Left	Left level IB
3	45	Female	3	1	2	III	Central	Bilateral level II
4	40	Male	2	1	0	I	Right	NA
5	76	Male	3	3	1	III	Left	Left RP, level II
6	55	Male	3	1	1	II	Right	Right level II

Abbreviations: WHO: World Health Organization; RP: retropharyngeal.

**Table 2:** Summary of differences in doses to vertebral arteries between intensity modulated proton therapy and volumetric modulated arc therapy plans.

	IMPT	VMAT	Paired Differences	*p* Value
Mean dose, Gy (RBE)				<0.001
Mean ± SD	31.6 ± 4.29	48.79 ± 2.5	17.19 ± 4.04	
Median (min to max)	31.99 (23.55 to 37.47)	48.91 (44.32 to 52.62)	17.27 (11.33 to 23.49)	
V10 (%)				0.001
Mean ± SD	89.3 ± 8.7	100.0 ± 0.0	10.7 ± 8.7	
Median (min to max)	90.6 (70.5 to 99.4)	100.0 (100.0 to 100.0)	9.4 (0.7 to 29.5)	
V20 (%)				<0.001
Mean ± SD	63.1 ± 9.3	99.9 ± 0.3	36.8 ± 9.5	
Median (min to max)	61.5 (47.9 to 77.9)	100.0 (98.9 to 100.0)	38.5 (21.0 to 52.1)	
V30 (%)				<0.001
Mean ± SD	50.2 ± 9.7	98.5 ± 2.3	48.3 ± 10.3	
Median (min to max)	51.3 (29.9 to 61.5)	99.9 (93.4 to 100.0)	45.4 (34.6 to 69.1)	
V40 (%)				<0.001
Mean ± SD	34.6 ± 9.5	86.6 ± 8.2	52.0 ± 12.6	
Median (min to max)	37.3 (14.6 to 48.9)	85.9 (75.1 to 100.0)	54.5 (32.8 to 70.6)	
V50 (%)				<0.001
Mean ± SD	22.4 ± 9.6	48.1 ± 14.1	25.7 ± 16.0	
Median (min to max)	21.0 (10.9 to 41.9)	53.4 (22.5 to 69.4)	31.0 (3.0 to 44.3)	
V60 (%)				0.608
Mean ± SD	6.5 ± 6.8	7.2 ± 7.6	0.7 ± 4.4	
Median (min to max)	4.6 (0 to 21.22)	5.2 (0 to 27.3)	0.3 (−9.0 to 6.0)	
V70 (%)				0.049
Mean ± SD	0.8 ± 1.2	0.2 ± 0.5	−0.6 ± 0.9	
Median (min to max)	0 (0 to 2.8)	0 (0 to1.6)	0 (−2.3 to 0.1)	

Abbreviations: IMPT: intensity modulated proton therapy; VMAT: volumetric modulated arc therapy; Gy (RBE): gray (relative biological effectiveness).

**Table 3:** Summary of differences in doses to clinical target volumes between intensity modulated proton therapy and volumetric modulated arc therapy.

	IMPT, Median (Range)	VMAT, Median (Range)	*p* Value
CTV_6996_			
Coverage (%)	99.5 (99.2−100.0)	99.8 (98.9−100.0)	0.776
Mean dose, Gy (RBE)	72.29 (66.75−73.39)	72.54 (68.84−72.84)	0.422
D5%, Gy (RBE)	73.90 (72.50−75.72)	74.41 (73.97−74.93)	0.370
D95%, Gy (RBE)	71.19 (70.69−71.36)	70.97 (70.6−71.33)	0.366
CTV_5940_			
Coverage (%)	99.8 (99.2−100.0)	99.9 (99.3−100.0)	0.213
Mean dose, Gy (RBE)	69.68 (68.99−71.13)	69.04 (68.72−70.2)	0.008
D5%, Gy (RBE)	73.54 (72.53−75.35)	73.95 (73.57−74.55)	0.423
D95%, Gy (RBE)	61.75 (60.86−63.21)	61.25 (61.16−61.73)	0.167
CTV_5280_			
Coverage (%)	99.9 (99.8−100.0)	99.9 (99.8−100.0)	0.091
Mean dose, Gy (RBE)	66.69 (65.13−67.89)	66.14 (64.16−66.81)	0.022
D5%, Gy (RBE)	73.39 (72.29−75.16)	73.74 (73.16−74.32)	0.596
D95%, Gy (RBE)	54.58 (53.66−55.01)	54.28 (54.09−54.54)	0.615

Abbreviations: IMPT: intensity modulated proton therapy; VMAT: volumetric modulated arc therapy; CTV: clinical target volume; Gy (RBE): gray (relative biological effectiveness); D5%: the dose received by the 5% volume; D95%: the dose received by the 95% volume.

## Data Availability

The data presented in this study are available upon request from the corresponding author. The data are not publicly available due to ethical restrictions.
